# Child internalizing and externalizing behaviors: Interplay between maternal depressive symptoms and child inhibitory control

**DOI:** 10.1002/jcv2.12107

**Published:** 2022-10-12

**Authors:** Tone K. Hermansen, Kari E. Syrstad, Espen Røysamb, Annika M. D. Melinder

**Affiliations:** ^1^ Department of Psychology University of Oslo Oslo Norway; ^2^ Norwegian Institute of Public Health Oslo Norway

**Keywords:** CBCL, child behavior, inhibitory control, maternal depressive symptoms, MoBa

## Abstract

Maternal depression is a risk factor for child internalizing and externalizing behaviors. Aiming to investigate the moderating role of child inhibitory control on this relationship, we invited a sub‐sample of dyads from the Norwegian Mother, Father, and Child Cohort study (MoBa) for a lab‐based assessment (*N* = 92, *M*_age_ = 68 months, Range = 59–80, 50% girls). Maternal depression was assessed using the Beck Depression Inventory (BDI‐II), while child behaviors were measured using the Child Behavior Check List, and inhibitory control using a child friendly version of the Flanker‐task. As expected, higher levels of concurrent maternal depressive symptoms predicted higher levels of child internalizing and externalizing behaviors. Importantly, and in line with our predictions, child inhibitory control moderated the association. Lower levels of inhibitory control predicted a stronger association between concurrent maternal depressive symptoms and child behavioral outcomes. The results support prior research suggesting that concurrent maternal depression poses a risk for child development, and highlight that children with lower levels of inhibitory control are more vulnerable to negative environmental influences. These findings contribute to our understanding of the complexity of parental mental health issues on child development and suggest avenues for personalized treatment programs for families and children at risk.

## Graphical abstract

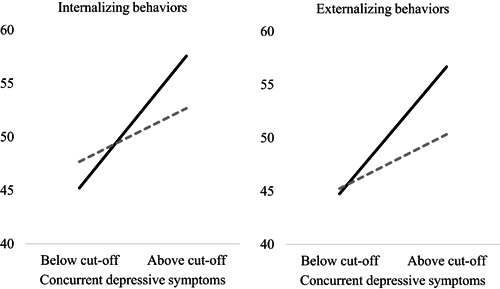

Maternal depression is a known risk factor for child internalizing and externalizing behaviors. In this study, we investigated the moderating role of child inhibitory control on the association between maternal depressive symptoms and child internalizing and externalizing behaviors. As expected, lower levels of inhibitory control predicted a stronger association between concurrent maternal depressive symptoms and child behavioral outcomes.

**Key points**

- Exposure to maternal depressive symptoms during early childhood is typically associated with negative behavioral outcomes.
- Prior work suggests individual differences in children's vulnerability to the negative effects of maternal depression.
- We investigate whether child inhibitory control may moderate association between maternal depressive symptoms and child internalizing and externalizing behaviors.
- In line with prior research, higher levels of maternal depressive symptoms corresponded with higher levels of child negative internalizing and externalizing behaviors.
- In line with our predictions, lower levels of inhibitory control predicted a stronger association between concurrent maternal depressive symptoms and child negative behavioral outcomes.
- Identifying early predictors of potential moderators of vulnerability or resilience is crucial for the successful development of personalized treatment programs.

## CHILD INTERNALIZING AND EXTERNALIZING BEHAVIORS: INTERPLAY BETWEEN MATERNAL DEPRESSIVE SYMPTOMS AND CHILD INHIBITORY CONTROL

Maternal depression during early childhood is a well‐known risk factor for later child development of internalizing and externalizing problems (Goodman & Gotlib, 1999; Goodman et al., 2011; van Santvoort et al., 2015). However, the negative effects of maternal depressive symptoms on child behavior varies greatly in severity (Beardslee et al., 2011). This suggests that there may be individual differences in children's vulnerability to the stressor of maternal depression. Indeed, several studies show that child cognitive capacities play a key role in the development of a range of problem behaviors (Gorka et al., 2015; Flouri et al., 2016). Moreover, as recent research has emphasized that not only early, but also later exposure to maternal depression may negatively impact children's cognitive and behavioral development (Gjerde et al., 2017; Lahti et al., 2017), the present study investigates the association between concurrent maternal depressive symptoms and preschool children's internalizing and externalizing behaviors, as well as the moderating effect of child inhibitory control on this association. Identifying early predictors of deviant development and potential moderators of vulnerability or resilience is crucial for the successful development of personalized treatment programs (Goodman & Garber, 2017).

### Maternal depression and its impact on early child development

Independent effects of prenatal, postnatal, and later concurrent maternal depressive symptoms on child internalizing and externalizing problems have all been reported (Gjerde et al., 2017; Lahti et al., 2017). Aiming to understand the transfer of risk from depressed mothers to their child, Goodman and Gotlib (1999) describe how maternal depression poses a risk to child development through mediators such as genetics and neuroregulatory mechanisms. On the one hand, depression is associated with a genetic predisposition (for a review, see Shadrina et al., 2018), a disposition that may be inherited by the child and as such increasing their risk of early internalizing behaviors and later depression. Guided by the notion that early exposure to adversity may have more negative effects on later child development, it has traditionally been more common to study the effects of maternal depression during pre‐ and postnatal stages (O’Connor et al., 2016). However, concurrent maternal depressive symptoms have been increasingly recognized as a significant risk factor (Eberhard‐Gran et al., 2004; Woolhouse et al., 2015). For example, being primary caretakers, parents influence their children's thoughts and behaviors indirectly through their own modelled behavior (Denham et al., 1997), as well as through their active regulation of their child's emotional responses (for a meta‐analysis, see Zimmer‐Gembeck et al., 2022). Mothers who suffer from depressive episodes may struggle to connect with their child during early social interactions (Harnish et al., 1995) and tend to respond less adequately to their child's behaviors (Coyne & Thompson, 2011; Dix et al., 2004; Shaw et al., 2006), providing the child with less tools to eventually regulate their own behaviors adequately. Indeed, a set of recent population based, longitudinal studies suggest that concurrent maternal depressive symptoms either partially mediates (Lahti et al., 2017), or fully moderates (Closa‐Monasterolo et al., 2017) the association between a mother's early depressive symptoms and later child behavioral problems. For example, Closa‐Monasterolo et al. (2017) reported that children's internalizing and externalizing behaviors at age eight were highest when mothers reported both postnatal depression and current mental health problems. Children exposed to mothers with only postnatal‐ but not later mental health problems did not differ from children without exposure to maternal depression (Closa‐Monasterolo et al., 2017). Controlling for gender, ethnicity, and socio‐economic status, Flouri et al. (2016) also noted clear associations between concurrent maternal depression and both child internalizing and externalizing problems. In sum, these studies suggest a partially additive effect of perinatal and later concurrent depression, but the effect seems driven by later concurrent depressive symptoms.

### Child characteristics and the potentially moderating role of inhibitory control

In their integrative model of the transfer of risk from depressed mothers to their child, Goodman and Gotlib (1999) describe the importance of transactional relationships between individual characteristics and environmental influences during child development. According to this model, there are a set of moderators that may affect the strength of the relationship between maternal depression and negative child outcomes. For example, Goodman and Gotlib (1999) hypothesized that children's cognitive skills would moderate the effects of exposure to maternal depression. The rationale being that children's ability to regulate their thoughts and behavior during everyday interactions hinges on the development of higher order cognitive control capacities often referred to as executive functions—a set of cognitive functions enabling adaptive responses to new or ambiguous situations (Diamond, 2013), encompassing inhibitory control, working memory, and cognitive flexibility (Miyake et al., 2000). Apart from a few recent studies, this hypothesis has remained largely untested.

Trying to uncover potential mechanisms of the association between maternal depression and child behavioral problems, two recent studies have conducted lab‐based assessments to investigate the role of child cognitive functions in relation to maternal depression and child internalizing and externalizing behaviors. For example, Flouri et al. (2016) found that children with depressed mothers *and* working memory challenges may be especially vulnerable to developing internalizing and externalizing problems. Following children from preschool age to the end of primary school, they found that working memory deficits moderated the association between maternal depression and both child internalizing and externalizing problems. The associations were stronger for children with lower working memory capacity (Flouri et al., 2016). This study thus represents an important new step towards understanding the mechanisms subserving risk and resilience and the impact of individual differences in cognitive development. However, while working memory may aid children's ability to remember what they should do in various situations etc., behaving or thinking in a socially adequate manner also entails the assertion of some form of control over impeding thoughts or emotions. As such, inhibitory control—the ability to resist impulses and to regulate thoughts, behaviors, and emotions (Diamond, 2013)—could be an additional factor closely tied to children's ability to avoid ruminating thoughts as well as unwanted and externalizing behaviors (Hofmann et al., 2012). Inhibitory control has traditionally been measured using a range of different tasks, and the age of onset and/or mastery of such skills during early development thus varies considerably with the demands of the task (for a review, see Garon et al., 2008). For example, infants as young as 4 months of age are able to inhibit a reflexive eye movement when presented with a simple response inhibition task, but are not able to combine the inhibition of one response with the activation of another until they reach 12 months of age (Scerif et al., 2005). Presented with more complex inhibition tasks demanding the regulation of behavior based on an inferred rule, which would be the equivalent of a more real‐world situation and demanding the combination of verbal and/or working memory skills, children continue to show great improvements across a range of tasks from ages 2 to 5 before performance begins to stabilize around age 7 (e.g., Carlson, 2005; Kochanska et al., 1996, 1997; Rothbart et al., 2003; Rueda, Posner, et al., 2004).

Inhibitory control is positively related to psychosocial adjustment (Kochanska et al., 1997), with greater inhibitory control traditionally being associated with greater internalizing problems (e.g., Joormann & Gotlib, 2010; Snyder, 2013), and lower inhibitory control typically being related to more externalizing problems (e.g., Olson et al., 2011; Utendale et al., 2011; Utendale & Hastings, 2011). However, given the heterogeneity in the tasks assessing inhibitory control across these studies, recent research suggests that these conclusions may require further nuancing (Berger & Buttelmann, 2022). Rather than different levels of inhibitory control predicting a certain behavioral problem, variations in children's inhibitory control could serve to explain the commonly observed link between environmental influences and behavioral problems (van Dijk et al., 2017). Support for this notion comes from recent research showing that inhibitory control moderates the association between other negative environmental influences and child behaviors (Hogye et al., 2022; Yu et al., 2018). For example, assessing the relationship between harsh parenting and child involvement in bullying, Hogye et al. (2022) found that girls who struggle with inhibitory control are more negatively affected by mothers harsh parenting in the sense that they are more likely to act as the perpetrator. Children with low levels of inhibitory control have also been found to be more vulnerable to the implications of physical punishment—as reflected in increased externalizing problems (Yu et al., 2018). Similarly, studies have found a moderating role of the closely related concept *effortful control*—the attentional and inhibitory mechanism that facilitates the inhibition of a dominant response (Rothbart et al., 2000)—on the relationship between contextual factors such as maternal mental health and child adjustment problems (Lengua et al., 2008), as well as on the relationship between mothers' depressive symptoms and child externalizing behaviors (Choe et al., 2014). Moreover, Wang and Dix (2017) found that the relationship between maternal depressive symptoms in infancy and poor socio‐emotional adjustment later in childhood was mediated by child executive functions.

### The present study

In this study, we investigate the association between concurrent maternal depressive symptoms on preschool children's internalizing and externalizing behaviors, and the moderating role of child inhibitory control—focusing on an age in which children's regulation strategies are still maturing yet can be reliably measured using the Flanker task (e.g., Rueda, Fan, et al., 2004). In line with prior research (Closa‐Monasterolo et al., 2017; Gjerde et al., 2017), we expected an association between concurrent maternal depressive symptoms and both internalizing and externalizing problems in preschool children. In addition, building on Goodman and Gotlib's (1999) notion that child cognitive development moderates the effects of adverse exposures during childhood, we expected children with poorer inhibitory control to be more vulnerable to the negative behavioral effects associated with exposure to maternal depression. We expected that the association between concurrent maternal depressive symptoms and both child internalizing and externalizing problems would be stronger for children with low inhibitory control.

## METHODS

### Participants and attrition

The current study is part of a larger project on child development following early exposure to maternal depressive symptoms. Participants were recruited to the overarching project from The Norwegian Mother, Father and Child Cohort Study (MoBa), a prospective population‐based pregnancy cohort study initiated by the Norwegian Institute of Public Health (Magnus et al., 2006, 2016), and for which participants were recruited in the time‐period 1999–2008. From this population‐based cohort study, a sub‐sample of women (*N* = 667) were invited to participate in an in‐depth lab‐based testing together with their child, based on a set of pre‐defined criteria regarding maternal mental health during and after pregnancy. Following this invitation, approximately 15% of the eligible women (*N* = 103) agreed to participate (for more details on the full sampling procedure and dropout analyses see Hermansen et al., 2016). Both MoBa and the current study has been approved by the Regional Committee for Medical Research Ethics (ref. 2013/794) and data protection offices (ref. 153,058). The study is based on version eight of the quality‐assured data files released for research (Magnus et al., 2016).

The effect sizes for the present study were expected to be small (Goodman et al., 2011). Thus, given a small effect size (Cohen's *d* = 0.20), and a significance level of *p* < 0.05, sample size should be at least *N* = 88 for a true effect to occur, leaving the available sample suitable for the planned analyses. The power estimate is derived using G*Power, with parameters specified for a regression model with three predictor variables (i.e., maternal depressive symptoms, child inhibitory control, and their interaction term) (Faul et al., 2007).

### Procedure

Parents agreeing to participate signed an informed consent form, on behalf of themselves and their child. To ensure children's rights to withstand from participating against their will (NESH, 2016), a child friendly version of the general information pamphlet was given to the parents prior to their participation. This pamphlet aimed to convey a pedagogically sound presentation of the project and testing situation, better suitable for young children than the formal consent form administered to parents. Following consent, children who had reached 5–6 years of age were invited together with their mothers for an in‐depth assessment in our lab at a time of their convenience. The assessment involved the children performing, amongst other, a child‐friendly version of the Flanker task. The mothers filled out Child Behavior Checklist (Child Behavior Check List) and Beck Depression Inventory II (BDI‐II) during this test session.

### Measures

#### Demographic information and prior maternal symptoms of anxiety and depression

Pregnancy‐related and demographic information was collected by researchers from the MoBa cohort study through a series of questionnaires (Version 8; Magnus et al., 2016). For the present study, only a subset of this data is included. To get a sense of sample demographics, we gathered information on relationship status (married/cohabitant vs. single/divorced), and maternal education level (general vs. higher education).

To generate an overall score of prior maternal symptomology, we also acquired data on prior maternal emotional distress using a short form of the Hopkins symptom checklist (SCL‐25), the SCL‐SF, during the first trimester (five items), third trimester (eight items), and six months postpartum (eight items) (Tambs & Moun, 1993; Tambs & Røysamb, 2014). Each item on the SCL is scored as 1 = “not bothered”, 2 = “a little bothered”, 3 = “quite bothered”, or 4 = “very bothered”. The average item score, calculated by dividing the total score of the number of items answered (ranging between 1 and 4), is used to reflect the level of prior symptoms of anxiety and depression, with values above 1.75 considered as a valid predictor of mental distress as assessed by clinical interviews (Sandanger et al., 1998; Strand et al., 2003). Finally, we generated an overall composite score of prior maternal symptoms by averaging the scores from the three time points during and after pregnancy, with a high score reflecting higher symptoms levels.

#### Concurrent maternal depressive symptoms

Measures of concurrent maternal depressive symptoms were assessed at the time of testing using the Beck Depression Inventory (BDI‐II; Beck, 1993; Beck et al., 1961), a widely used self‐report screening instrument of depressive symptoms, showing good reliability and validity (Siqveland & Kornør, 2011; Sprinkle et al., 2002; Storch et al., 2004). The questionnaire is composed of 21 groups of four statements, where the respondent selects the statement best describing how they have felt in the past week. The scale is cumulative, giving a total score between 0 and 63, with a score of 14–19 indicating mild depression, 20–28 moderate depression, and 29–63 severe depression (Siqveland & Kornør, 2011).

#### Child internalizing and externalizing problems

Child Behavior Checklist (CBCL; Achenbach & Rescorla, 2000) is one of the most frequently used rating scales for measuring child and adolescent internalizing and externalizing problems for research purposes (Achenbach et al., 2016), demonstrating high reliability and validity (Achenbach & Rescorla, 2000; Dutra et al., 2004). In the present study, parents were asked to fill out either the preschool version (1½ ‐ 5 years) or school version (6–18 years), depending on the child's age at the time of assessment. In line with prior reports, internal consistency was high for both internalizing behaviors, *α* = 0.79, and externalizing behaviors, *α* = 0.81.

#### Child inhibitory control

The original Flanker task was developed by Eriksen and Eriksen (1974), and represents a measure of inhibitory control (Diamond, 2013). In order to create a child‐friendly version of the Flanker task, we replaced the traditional arrows used in the Flanker task with stimuli from the Attentional Network Task for children (Hermansen et al., 2016; Rueda, Fan, et al., 2004, Rueda, Posner, et al., 2004). The stimuli were presented using E‐Prime 2.0 software (Psychology Software Tools Inc., Sharpsburg, PA), and Windows XP professional, and shown on a 20‐in color LCD monitor (Flex Scan L768) with 1280 by 1024 in screen resolution and 32‐bit color quality. The child was seated approximately 45 cm from the monitor.

**FIGURE 1:**
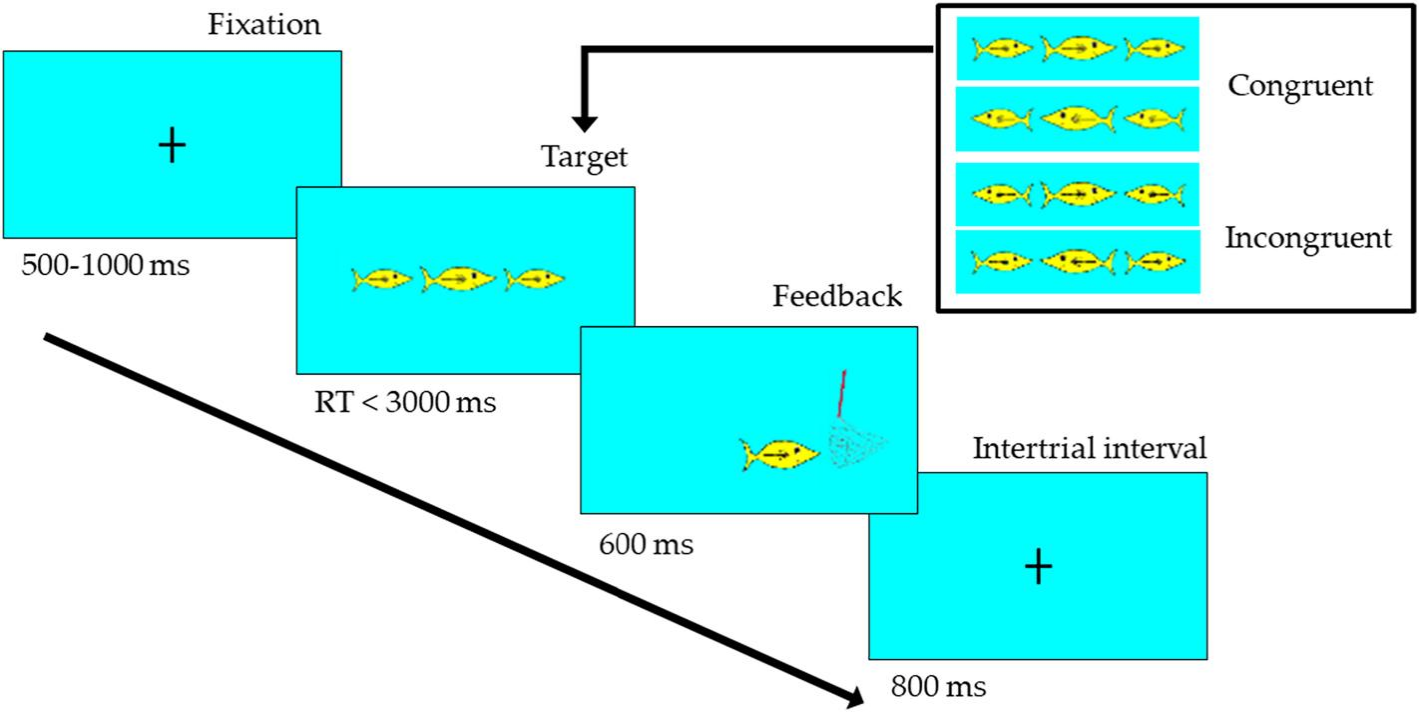
Illustration of the experimental procedure for the child‐friendly version of the Flanker‐task used in the present study

Seated in front of the monitor, children were told that they would play a computer game, where the central goal is to catch as many target animals as possible. To catch a target animal, children were instructed to pay attention to the direction the animal was moving, and to put up a net in front of the animal so as to catch it by pressing the arrow key corresponding to the animal's orientation. To challenge children's inhibitory control, the target animal was presented in the middle of a row of five other animals, where the four flankers—two on each side—acted as distractor stimulus (see Figure 1). On half of the trials the flankers were oriented in the same direction as the target animal (congruent trial), and in the other half of the trial, the flankers were oriented in the opposite direction to the target animal (incongruent trials). Congruent trials thus required children to keep the rules of the game in mind—tapping mainly into working memory capacities—and placed little demands on inhibitory control processes. The incongruent trials on the other hand, both required children to keep the rules in mind, and also stressed children's ability to ignore the dominating visual that the majority of the animals moved in the opposite direction to that of the target animal. Children performed a total of 240 trials, half of which were incongruent, and their responses were recorded in terms of accuracy and response times for each trial. In the present analysis, we used children's accuracy scores as the central measure of inhibitory control, as accuracy has been found to be a more sensitive measure than reaction times in young children (Diamond & Kirkham, 2007). To control for children's general performance and the tasks general demands on working memory, we computed a difference score by subtracting children's accuracy on the incongruent trials (as reflected in their percentage of correct responses) from their accuracy score on the congruent trials, and then reversed this score to enable a more intuitive interpretation of the score. That is, children with a large difference score show better performance on the incongruent trials relative to congruent trials, and thus indicates greater inhibitory control compared to children with a small difference score.

### Data inspection

#### Missing data

For the present study, the core variables of interest were derived through the lab‐based testing, focusing on maternal depressive symptoms at time of testing, and child behavioral problems and inhibitory control at age 5–6. Due to missing data on the main outcome variable (Child Behavior Checklist, *N* = 7), and on the measure of inhibitory control (Flanker task, *N* = 5), the final sample of the main analysis totaled 92 participants (Girls: *N* = 46, 50%).

#### Sample characteristics

At the time of birth, mother's mean age was 31.5 years (range = 19–44 years). At the time of testing, children's mean age was 5 years and 8 months (*M* = 68 months, range = 59–80 months). Most of the women were married or cohabiting with a partner at the time of initial assessment (*N* = 83, 90.22%), and only a minority reported being single or divorced (*N* = 5, 8.62%) (four missing values). The majority of women had also completed higher education (*N* = 60, 65.22%), with a minority reporting to have only basic schooling or less (*N* = 26, 28.26%) (six missing values). Information about the association between these demographic variables and the main variables of interest are presented as exploratory analyses in Supporting Information S1.

The MoBa‐study has previously reported that participating families mainly belong to resource rich families of high socio‐economic status, with an underrepresentation of single mothers (Nilsen et al., 2009). In an attempt to assess whether there may be any additional selection biases in the current sub‐sample, we analyzed selected demographic characteristics of participating and non‐participating dyads (e.g., maternal age, relationship status, maternal education). One‐way analysis of variance revealed no differences in age between participating (*M* = 31.40, *SD* = 4.83) and non‐participating women (*M* = 30.82, *SD* = 4.77), *F*(1,671) = 1.26, *p* = 0.261. Furthermore, Chi‐square analysis revealed no difference in the likelihood of being in a relationship between those who participated (94.9%) and not (97.4%), *X* ^*2*^(1) = 1.78, *p* = 0.182. There was no difference in the number of women having completed higher education among those who participated (70.8%) and not (68.1%), *X* ^*2*^(1) = 0.29, *p* = 0.593. Furthermore, given that this study aims to explore the relationship between different variables, on not the prevalence of a given phenomenon, we also explored possible differences between participating and nonparticipating dyads by assessing the association between the available demographic predictors and prior maternal symptomology (collapsing the scores from the three short forms of the SCL‐25, the SCL‐SF, administered during the first trimester, third trimester, and 6 months postpartum). Using standardized scores, we ran a series of hierarchical regressions where we used mothers composite SCL score as the dependent variable, and introduced the Demographic variable of interest (i.e., Maternal age, Relationship status, Maternal education) as the first step, before introducing Participation (Participating vs. Non‐participating), and finally the Participation BY ‘Demographic variable’ interaction term. The analyses revealed no significant interaction between Participation and Maternal age (*F*_*Δ*_(1, 657) = 0.33, *p* = 0.569), Participation and Relationship status (*F*_*Δ*_ (1637) = 0.23, *p* = 0.633), or Participation and Maternal education (*F*_*Δ*_(1, 612) = 3.72, *p* = 0.054). Note that these comparisons are made between the full sample of women who were eligible for inclusion and the sample who agreed to participate, some cases were later excluded to meet the inclusion criteria of the current investigation.

### Analytical plan

All data were analyzed using the statistical software package SPSS version 26 (SPSS Inc., Chicago, IL, USA). Complementary analyses are reported in Supporting Information S1. To aid interpretation of the regression analysis, all predictor scores were standardized before computing the interaction terms.

To assess the moderating role of child inhibitory control on the association between concurrent maternal depressive symptoms and child internalizing and externalizing behaviors we first ran a set of bivariate correlations assessing the overall associations between the variables of interest. Next, we ran a set of hierarchical regression analysis—one for each outcome measure. Common to both sets of regression models we first included concurrent maternal depressive symptoms as the main predictor of child outcome, before adding inhibitory control as a second step, and finally, the interaction term (Concurrent maternal depressive symptoms X Child inhibitory control) as a third step. To control for the possibility that prior exposure to maternal symptoms of anxiety and depression, as reflected in the SCL‐SF, was the driving force behind the observed effects, we replicated both sets of models including a composite score of prior maternal symptoms as a preliminary step in the analysis. We present these models in Supporting Information S1.

as they revealed no change in the moderating role of child inhibitory control on the relationship between concurrent maternal depressive symptoms and child behaviors. The Supporting Information S1 also present exploratory analyses in which child gender, relationship status, and maternal education are included as covariates—neither of which changed the moderating role of child inhibition in the main models presented below.

## RESULTS

### Measurement outcomes

As displayed in Table 1, the average levels of concurrent depressive symptoms were low, with approximately 14.1% (*N* = 15) of the women reporting concurrent depressive symptoms above the cut‐off of 14, indicating mild depression. Children's mean T‐scores for internalizing and externalizing problems are lower in this sample than in the normative sample (*M* = 50). Nonetheless, approximately 8.7% (*N* = 8) of the children scored above the cut‐off for borderline clinical scores (*T* > 65) on at least one of the scales. Children's performance on the Flanker task was generally high, although children's accuracy was significantly lower on the incongruent trials compared the congruent trials, *t*(91) = 4.43, *p* < 0.001 (see Table 1). In the following regression analyses, child inhibitory control is derived by subtracting child accuracy on incongruent trials from their accuracy on congruent trials, and then reversing the score. Thus, children with a large difference score show greater inhibitory control compared to children with a small difference score.

**TABLE 1:** Descriptive statistics of maternal depressive symptoms and child outcomes

	M	SD	Range
Concurrent maternal depressive symptoms	8.00	7.95	0–37
Child internalizing behavior	48.01	11.32	29–71
Child externalizing behavior	46.50	12.24	28–85
Flanker task			
Incongruent trial accuracy	0.90	0.12	0.33–1.00
Congruent trial accuracy	0.93	0.07	0.67–1.00
Child inhibitory control	0.52	0.08	0.01–0.66

### Preliminary analysis

Examining the associations between the main variables of interest, we first ran a set of bivariate correlations between maternal concurrent depressive symptoms, child internalizing and externalizing behaviors, and child inhibitory control (for details, see Table 2). As expected, the two outcome variables, internalizing and externalizing behaviors, were highly correlated. The predictor variable concurrent maternal depressive symptoms was significantly correlated with both outcome variables. Child inhibitory control showed a weak association with the other variables, although this was not statistically significant. However, based on theoretical assumptions, inhibitory control may still have a moderating effect on the association between concurrent maternal depressive symptoms and child internalizing or externalizing behaviors without having a direct correlation with the outcome. Thus, all variables were included in the following analysis examining whether children's scores of inhibitory control, moderates the observed association between maternal concurrent depressive symptom levels and child internalizing and externalizing behaviors.

**TABLE 2:** Correlations between maternal depressive symptoms and child outcomes

			1	2	3
1	Concurrent maternal depressive symptoms	*r*	1		
		*p*	.		
		*CI*	.		
2	Child internalizing behavior	*r*	0.44		
		*p*	<0.001		
		*CI*	[0.26, 0.59]		
3	Child externalizing behavior	*r*	0.37	0.72	
		*p*	<0.001	<0.001	
		*CI*	[0.12, 0.54]	[0.60, 0.81]	
4	Child inhibitory control	*r*	−0.04	0.09	−0.09
		*p*	0.642	0.379	0.387
		*CI*	[−0.25, 0.16]	[−0.11, 0.29]	[−0.29, 0.12]

*Note*: Correlations (*r*) reflect Pearson correlation coefficient, with significance (*p*) determined using 2‐tailed tests, and 95% confidence intervals (CI [LL, UL]) based on Fisher's r‐to‐z transformation.

### Internalizing behaviors

Assessing the moderating role of child inhibitory control on the association between concurrent maternal depressive symptoms and child internalizing behaviors, we ran a hierarchical regression analysis with concurrent maternal depressive symptoms entered first as the main predictor of child outcomes. As expected, this revealed an overall significant model *F* (1,90) = 22.01, *p* < 0.001, with concurrent maternal depressive symptoms explaining 19.6% of the variance in child internalizing behaviors. Increasing levels of concurrent maternal depressive symptoms is associated with an increase in child internalizing behaviors (see Table 3, Model 1). As a second step, after controlling for concurrent maternal depressive symptoms in step one, child inhibitory control was added to the model. While the model as a whole remained significant (Table 3, Model 2), child inhibitory control did not significantly increase the model's explained variance, *F*_Δ_(1,89) = 1.48, *p* = 0.227, *R* ^*2*^_Δ_ = 0.013. Finally, we added the interaction term (Concurrent maternal depressive symptoms X Child inhibitory control) as a third step. This resulted in an overall significant model (Table 3, Model 3), and revealed that the interaction explained an additional 4.1% of the variance in child internalizing behaviors, *F*_Δ_(1,88) = 4.76, *p* = 0.032, *R* ^*2*^_Δ_ = 0.041.

**TABLE 3:** Hierarchical regression models testing the moderating effect of child inhibitory control on the association between concurrent maternal depressive symptom levels and child internalizing behaviors

	Model 1 (*β* [SE], *p*)	Model 2 (*β* [SE], *p*)	Model 3 (*β* [SE], *p*)
Concurrent maternal depressive symptoms	0.44 [0.09], <0.001	0.45 [0.09], <0.001	0.42 [0.09], <0.001
Child inhibitory control		−0.12 [0.09], 0.227	−0.22 [0.10], 0.039
Concurrent maternal depressive symptoms X child inhibitory control			−0.23 [0.08], 0.032
*R* ^*2*^	0.196	0.210	0.250
Model df	90	89	88
Model *F*	22.01	11.80	9.79
*p*	<0.001	<0.001	<0.001

**FIGURE 2:**
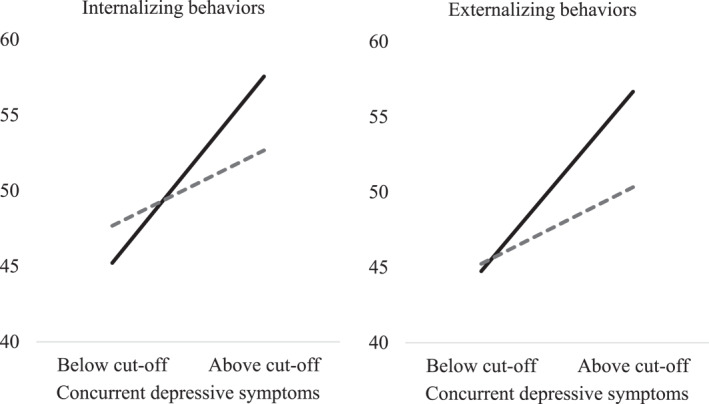
Illustration of child internalizing (left panel) and externalizing behaviors (right panel), as a function of concurrent maternal depressive symptom levels and child inhibitory control. Black lines represent children with lower than median inhibition (exposed to lower (*N* = 38) versus higher (*N* = 10) levels of maternal depressive symptoms), and dotted grey bars represent children with higher than median inhibition (exposed to lower (*N* = 42) versus higher (*N* = 8) levels of maternal depressive symptoms)

To clarify this interaction, which is displayed in the left panel of Figure 2, we examined the simple effect of maternal depressive symptoms on child internalizing behaviors for children with higher versus lower inhibitory control. Maternal depressive symptoms were grouped based on whether they scored above (*N* = 15) or below (*N* = 77) the cut‐off for mild depression. Because there are no standardized norms for assessing child inhibitory control using the Flanker task, we used a median‐split approach to distinguish between children who displayed higher (*N* = 47) versus lower (*N* = 45) inhibitory control. These post‐hoc analyses revealed a significant effect of maternal depressive symptoms on child internalizing behaviors only when child inhibition was low, *F*(1,44) = 9.08, *p* = 0.004. Children with lower levels of inhibitory control, displayed significantly more internalizing behaviors when maternal depressive symptom levels were high (*M* = 57.56, *SD* = 9.06), compared to low (*M* = 45.22, *SD* = 11.37). There was no effect of maternal depressive symptoms on child internalizing behaviors when child inhibition scores were above median*, F*(1,46) = 1.12, *p* = 0.296. Children with greater inhibitory control displayed no significant difference in internalizing behaviors when maternal depressive levels were high (*M* = 52.67, *SD* = 9.77), compared to low (*M* = 47.68, *SD* = 10.91).

### Externalizing behaviors

To investigate the moderating role of child inhibitory control on the association between concurrent maternal depressive symptoms and child externalizing behaviors we followed the same procedure as for the analysis of internalizing behaviors. Entered as the first step, concurrent maternal depressive symptoms revealed an overall significant model, *F*(1,90) = 14.45, *p* < 0.001, with concurrent maternal depressive symptoms explaining 13.8% of the variance in child externalizing behaviors. Increasing levels of concurrent maternal depressive symptoms is associated with an increase in child externalizing behaviors (see Table 4, Model 1). Adding child inhibitory control as a second step, the model as a whole remained significant (Table 4, Model 2), but child inhibitory control did not significantly increase the model's explained variance, *F*_Δ_(1,89) = 0.56, *p* = 0.458, *R* ^*2*^_Δ_ = 0.005. As the final step, we added the interaction term (Concurrent maternal depressive symptoms X Child inhibitory control). This resulted in an overall significant model (Table 4, Model 3), and revealed that the interaction term explained an additional 5.2% of the variance in child externalizing behaviors, *F*_Δ_(1,88) = 5.68, *p* = 0.019, *R* ^*2*^_Δ_ = 0.052.

**TABLE 4:** Hierarchical regression models testing the moderating effect of child inhibitory control on the association between maternal depressive symptoms and child externalizing behaviors

	Model 1 (*β* [SE], *p*)	Model 2 (*β* [SE], *p*)	Model 3 (*β* [SE], *p*)
Maternal symptoms	0.37 [0.10], <0.001	0.37 [0.10], <0.001	0.33 [0.10], <0.001
Child inhibitory control		−0.07 [0.10], 0.458	0.04 [0.11], 0.690
Maternal symptoms X child inhibitory control			−0.26 [0.09], 0.019
*R* ^*2*^	0.138	0.144	0.196
Model df	90	89	88
Model *F*	14.45	7.47	7.13
*p*	<0.001	<0.001	<0.001

To clarify this interaction, displayed in the right panel of Figure 2, we examined the simple effect of concurrent maternal depressive symptoms (above vs. below the cut‐off for mild depression) on child externalizing behaviors for children with high versus low inhibitory control using the same median split approach as above. The post‐hoc analyses revealed a significant effect of maternal depressive symptoms on child externalizing behaviors only when child inhibition was low, *F*(1,44) = 6.55, *p* = 0.014. Children with lower levels of inhibitory control, displayed significantly more externalizing behaviors when maternal depressive symptom levels were high (*M* = 56.67, *SD* = 12.61), compared to low (*M* = 44.75, *SD* = 12.47). There was no effect of maternal depressive symptoms on child externalizing behaviors when child inhibition scores were above median*, F* (1,46) = 1.07, *p* = 0.307. Children with greater inhibitory control, displayed no significant difference in externalizing behaviors when maternal depressive levels were high (*M* = 50.33, *SD* = 9.18), compared to low (*M* = 45.24, *SD* = 11.50).

Given that the current sample was recruited based on maternal mental health during and after pregnancy, as reflected in the SCL, this allowed us to control for the possibility that prior exposure to maternal symptoms of anxiety and depression was the driving force behind the observed effects of concurrent maternal depressive symptoms. To test this hypothesis, we replicated both sets of models including a composite score of prior maternal symptoms of anxiety and depression as a preliminary step in the analyses. However, while prior maternal symptoms of anxiety and depression were significantly correlated with later child internalizing and externalizing behaviors (*p*'s < 0.001), we found no change in the moderating role of child inhibitory control on the relationship between concurrent maternal depressive symptoms and child behaviors (for details, see Supporting Information S1).

We also ran additional analyses controlling for child gender, maternal education level, and relationship status. However, we found no change in the moderating role of child inhibitory control on the relationship between maternal concurrent depression and child behaviors when including these variables as covariates (see Supporting Information S1).

## DISCUSSION

In the current study, we investigated the moderating role of child inhibitory control on the association between concurrent maternal depressive symptoms and child internalizing and externalizing behaviors. As expected, the results revealed a clear association between concurrent maternal depressive symptoms and child internalizing and externalizing behaviors. Higher levels of concurrent maternal depressive symptoms corresponded with higher levels of child internalizing and externalizing behaviors. Importantly, and in line with our predictions, child inhibitory control moderated these associations. Lower levels of inhibitory control predicted a stronger association between concurrent maternal depressive symptoms and child negative internalizing and externalizing behavioral outcomes. Higher levels of inhibitory control could be considered a protective factor, but this is not clear from the current data. This supports the hypothesis that children with low inhibition skills are more vulnerable to the negative effects of maternal depression. In what follows, we discuss these findings in more detail. We highlight how our findings support emerging initiatives to assess not only the impact of maternal mental health during the pre‐and postnatal periods, but also extend the assessment further into childhood—assessing the impact of concurrent maternal mental health on child development. We also show how this study supports the hypothesis that child cognitive development moderates the impact of negative events in early childhood. Ultimately, the results indicate important avenues for further research on interventions targeting child cognitive development and the strengthening of executive functions.

### Maternal depressive symptoms and child behavioral problems

The bulk of prior research on maternal depressive symptoms has mainly been concerned with the potentially negative effects of pre‐ and postnatal exposure on child development. The current study supports recent work suggesting that also concurrent maternal depression poses a significant risk for child development (Closa‐Monasterolo et al., 2017; Gjerde et al., 2017; Lahti et al., 2017). In line with the findings of Gjerde et al. (2017), our results revealed a negative association between concurrent maternal depressive symptoms and child internalizing and externalizing problems. Even though our findings are in line with our initial predictions, the strength of this association is rather surprising given the low average levels of maternal depressive symptoms reported. Moreover, most of the women in the included sample had a higher education and lived with a partner, providing the child with an additional caregiver to seek support from when needed. These positive environmental factors likely buffer against some of the negative effects associated with exposure to maternal depression during childhood, leaving the observed findings even more striking.

One caveat to this interpretation is that the current sample was initially recruited as part of a larger study recruiting participants based on prior reports of anxiety and depressive symptoms during pregnancy (Hermansen et al., 2016). Thus, while the women on average reported relatively low levels of concurrent depressive symptoms, some of those who reported concurrent symptoms also reported depressive symptoms during pregnancy. One could therefore speculate whether the observed negative effects of maternal mental health on child behavior could be reflective of a strong genetic predisposition to depression (Sullivan et al., 2000), or to prolonged rather than concurrent exposure. Unfortunately, our sample size limited our analytical opportunities to run more fine‐grained path‐analysis delineating the full impact of prior depressive episodes. However, simple control analyses presented in the Supporting Information S1 indicate that although prior maternal symptoms of anxiety and depression is a significant risk of later child behaviors, particularly for externalizing problems, the moderating effect by child inhibitory control on the relationship between concurrent maternal depressive symptoms and child behaviors remained even when prior exposure was controlled for.

### The role of inhibitory control in explaining child behavioral problems

Studies assessing the mechanisms of the transferred risk of maternal mental health on child internalizing and externalizing behaviors have previously assessed various parent‐related mediators of this association, such as the impact of increased parental stress (e.g., Tsotsi et al., 2019), reduced parental sensitivity (Wang & Dix, 2017), or increased socio‐economic and environmental risk factors (e.g., Lengua et al., 2008). These findings highlight the importance of increasing social support and enhancing caregiver sensitivity among women who suffer from mental health issues, indicating important avenues for intervention on behalf of the parent. However, considering the continuous interplay between child characteristics and the quality of social support in their immediate environment (Sameroff, 2009), we wanted to examine how an individual child characteristic—such as inhibitory control—affect the strength and/or direction of the relationship between maternal depressive symptoms and child internalizing and externalizing behaviors.

Increased inhibitory control has typically been associated with a greater risk of more internalizing behaviors, and less externalizing problems (e.g., Olson et al., 2011; Utendale and Hastings (2011), Utendale et al., 2011). However, rather than having a differential effect on the two outcome variables, our analyses revealed that children with lower levels of inhibitory control were more vulnerable to the negative effects of concurrent maternal depressive symptoms in terms of both internalizing and externalizing behaviors. These findings are in line with the hypothesis that child cognitive functions may moderate the negative developmental effects typically associated with maternal mental health problems (Goodman & Gotlib, 1999; van Dijk et al., 2017). In line with the model's predictions, we found that children who struggle to regulate their thoughts and behaviors, displaying poorer inhibitory control, were more vulnerable to the negative effects of maternal depression than children with more well‐developed inhibitory skills. Importantly, and central to our interpretation of the findings, child inhibitory control was not significantly correlated with maternal depressive symptoms, or with internalizing or externalizing behaviors. The relevance of child inhibitory control was only evident when going beyond main effects, investigating how inhibition interacts with concurrent maternal depressive symptoms to predict child behaviors. These findings highlight the importance of investigating individual differences in the vulnerability to behavioral problems following exposure to atypical rearing environments.

Although no prior study has to our knowledge investigated the role of inhibitory control on the relationship between maternal depression and child behavioral problems, there are several similarities between our study and that of prior work on the moderating role of inhibitory control on the association between other negative environmental influence and child behaviors (Hogye et al., 2022; Yu et al., 2018). There is also overlap with research on other related constructs such as working memory and effortful control. For example, the moderating effect of inhibitory control is in line with recent work presented by Flouri et al. (2016)—uncovering a moderating role of working memory (e.g., another component of executive functions) on the association between maternal mental health and child behavioral problems. Specifically, Flouri et al. (2016) showed that children with lower scores on working memory were more vulnerable to the negative effects of maternal depression on child behaviors. With the current study focusing on the difference score between congruent and incongruent trials, we are able to further tease apart the mechanisms at play—with preliminary indications that inhibitory control may have an impact beyond that of working memory. This interpretation coincides with empirical investigations of children's effortful control—a construct referring to more overarching self‐regulatory functions similar to executive functions (Choe et al., 2014). Choe et al. (2014) found that children's effortful control at age 3, moderated the association between maternal depressive symptoms and child externalizing behaviors at age 10. Relatedly, Tsotsi et al. (2019) reported that self‐regulation skills at 3.5 years of age moderated the indirect effect of maternal trait anxiety, through parenting stress, on child externalizing behaviors at age 4. While Tsotsi et al. (2019) found no moderation effect of self‐regulation on child internalizing behaviors, others have reported effortful control to moderate the association between other maternal risk factors and internalizing behaviors in 8–12 year old children (Lengua et al., 2008). These slight discrepancies between earlier findings relating to internalizing problems, may stem from differences in the operationalization and assessment of child regulatory skills, as well as the nature of internalizing behaviors itself. For example, as Tsotsi et al. (2019) point out, they mainly assessed children's behavioral self‐regulation using the Snack‐Sticker Delay task, a task tapping intro constructs more closely linked to externalizing than internalizing behaviors, such as increased impulsivity. In the current study, we assessed inhibitory control using the Flanker task—a task demanding a broader range of control functions as it requires both the ability to inhibit impulsive or prepotent responses as well as engaging inhibitory resources to deflect attention from the flanker stimuli. Furthermore, assessing internalizing problems in young preschoolers is challenging as children at this age are not always capable of expressing their inner workings, or may display externalizing behaviors for issues that are inherently issues of internalization (Pavuluri et al., 1996). Previous studies have indicated that preschool teachers have difficulty detecting internalizing problems at the youngest ages (Bulotsky‐Shearer & Fantuzzo, 2004), and it is not unlikely that parents may also struggle to both notice or distinguish between different forms of behavioral problems. This hypothesis is supported by the high correlation observed between internalizing and externalizing behavior both in the current work, as well as in that of Tsotsi et al. (2019).

In a prior study, Wang and Dix (2017) reported a mediating role of child executive functions on the association between maternal depressive symptoms during child infancy and children's later socioemotional adjustment. They found that child executive functions were negatively related to maternal depressive symptoms during the child's first 2 years, and that this in turn affected later socioemotional development. This association is in line with prior work indicating a negative correlation between maternal depression and child executive functions in preschool children (Hughes, 2011; Hughes et al., 2013). It is unclear why such a mediating effect is not found the present study, but the limited sample size of the current study could be a contributing factor to such discrepancies. Future studies will be necessary to further examine the mechanisms with which maternal depression affects child cognitive and behavioral development.

### Practical implications

The observed negative effect of maternal depressive symptoms on child negative behaviors among children with lower levels of cognitive control, mainly highlights a vulnerating factor, but also hints at a potential protective factor. In the current sample, children who displayed stronger inhibitory skills were not affected by maternal depressive symptoms, as measured by the behavioral outcome scales. This resembles work from resilience researchers, showing that general executive functions are key protective factors for children growing up in contexts of severe adversity (Blair & Raver, 2012; Diamond et al., 2007; Sapienza & Masten, 2011). In other words, if replicated through further research, the role of child inhibitory control as a moderator of the association between maternal depression and child problem behaviors provides a promising route for interventions with a more dyadic focus—targeting individual child characteristics in addition to parent training and psychoeducation. Existing work has indicated that it is possible to train executive functions in young children (Diamond et al., 2007; Klingberg et al., 2005; Pandey et al., 2018). However, while these findings are promising, the generalizability of the results remain controversial (for a discussion see Shipstead et al., 2010; Thorell et al., 2009). Further research assessing the beneficial effects of preschool programs ability improve executive functions in young children are therefore necessary, taking into account the challenge of generalizing very specific skills acquired through controlled training settings to more variable everyday situations. If successful, such programs may provide cost‐effective child directed interventions that can enhance skills immediately relevant for academic achievements (Diamond et al., 2007), and also supplement existing parent‐directed interventions working to strengthening the child's social environment (Goodman & Garber, 2017).

### Strengths and limitations

In the present study, we found strong associations between concurrent maternal depressive symptoms, and negative outcomes on both child internalizing and externalizing behaviors. In addition, and central to our key hypothesis, we found small to medium moderations effects of inhibitory control on these associations. These associations were in line with our initial predictions, and with similar studies in related fields. Moreover, an important strength of this work is that we circumvent the concern that depressed mothers provide inaccurate reports of their child's cognitive development. For example, depressive symptoms may alter the mothers' view of their child, perhaps because they find even typical child behavior more exhausting than other parents do, reducing the validity of their assessment (Müller et al., 2011). By utilizing a computerized and child‐friendly version of the Flanker task we could measure child inhibitory control directly, independent of maternal responses. The lack of a significant association between maternal depressive symptoms and child inhibition scores, yet clear moderation effects of inhibitory control on the association between maternal mental health and child behaviors, suggests a robust effect. That said, some limitations apply.

First, the socio‐demographic profile of the invited sample, and that of MoBa in general (Nilsen et al., 2009), reflects a group with relatively high levels of education and stable partnerships. These features challenge the interpretative power of our findings concerning prevalence. Fortunately, associations between selected variables and the generalization of these are found to be less vulnerable to sample effects (Nilsen et al., 2009). Comparing data from MoBa and the national birth registry, Nilsen et al. (2009) investigated differences in prevalence‐ and association measures of well‐known risks/associations such as the link between maternal smoking and low birthweight. The researchers concluded that prevalence estimates of exposures and outcomes, but not estimates of exposure‐outcome associations, are biased due to self‐selection (Nilsen et al., 2009).

Second, the average levels of maternal depressive symptoms in the current sample is low, children's average behavioral scores were within the normal ranges, and children's average inhibition scores was close to ceiling levels. Thus, it may be difficult to compare our results with those observed among parents and children with more extreme scores on either measure. For example, it is unknown whether the moderation effect of inhibition would be stronger in dyads were maternal depression is more severe and child behavioral problems more prominent, or conversely, whether it's moderating role may disappear. However, given that we find small, albeit significant indications of moderation in a fairly well performing sample such as the present, highlights the significance of child cognitive control in the face of adversity and risk of developmental challenges.

Finally, while the results of our moderation analyses are primarily in line with the diathesis stress model, positing that children with an existing vulnerability (i.e., poor inhibitory control) may be more prone to poor developmental outcomes if exposed to negative environmental influences (Sameroff, 2009), the current study does not rule out the possibility that these children could also be more sensitive to positive environments (Boyce et al., 1995), thus having greater receptivity to intervention.

## CONCLUDING REMARKS

We investigated the moderating role of child inhibitory control on the association between maternal depressive symptoms on child internalizing and externalizing behaviors. In line with recent research, the results revealed a clear association between maternal depressive symptoms and child internalizing and externalizing behaviors. Higher levels of maternal depressive symptoms corresponded with higher levels of child negative internalizing and externalizing behaviors. Importantly, and in line with our predictions, child inhibitory control moderated these associations. Lower levels of inhibitory control predicted a stronger association between concurrent maternal depressive symptoms and child negative behavioral outcomes. This supports the hypothesis that children with low inhibitory control are more vulnerable to the negative effects of maternal depression. These findings are important for our understanding of the complex and transactional relationship between maternal mental health and child development and suggest promising avenues for the development of personalized treatment options.

## AUTHOR CONTRIBUTIONS

**Tone K. Hermansen**: Conceptualization; Data curation; Analysis; Investigation; Methodology; Project administration; Software; visualization; Writing – original draft; Writing – review & editing. **Kari E. Syrstad**: Conceptualization; Analysis; Investigation; Methodology; Writing – review & editing. **Espen Røysamb**: Conceptualization; Data curation; Investigation; Methodology; Project administration; Supervision; Writing – review & editing. **Annika M. D. Melinder**: Conceptualization; Data curation; Funding acquisition; Investigation; Methodology; Project administration; Resources; Supervision; Writing – review & editing.

## CONFLICTS OF INTEREST

The authors have declared that they have no competing or potential conflicts of interest.

## ETHICAL CONSIDERATIONS

Both MoBa and the current study has been approved by the Regional Committee for Medical Research Ethics (ref. 2013/794) and data protection offices (ref. 153058).

## Supporting information

Supplementary Material S1

## Data Availability

Data from the Norwegian Mother, Father and Child Cohort Study and the Medical Birth Registry of Norway used in this study are managed by the national health register holders in Norway (Norwegian Institute of public health) and can be made available to researchers, provided approval from the Regional Committees for Medical and Health Research Ethics (REC), compliance with the European Union's General Data Protection Regulation and approval from the data owners. The consent given by the participants does not open for storage of data on an individual level in repositories or journals. Researchers who want access to data sets for replication should apply through helsedata.no. Access to data sets requires approval from The Regional Committee for Medical and Health Research Ethics in Norway and an agreement with MoBa.
