# Supplementary material for: Child internalizing and externalizing behaviors: Interplay between maternal depressive symptoms and child inhibitory control
Source: JCPP Adv. 2022 Oct 12;2(4):e12107. doi: 10.1002/jcv2.12107 (PMC10242951; doi:10.1002/jcv2.12107)
Supplement: Supplementary file 1 — Supplementary Material S1 [file JCV2-2-e12107-s001.docx]

**Supporting Information**

Measures of maternal symptoms of anxiety and depression during and immediately after pregnancy were acquired through the short form of the symptom checklist (SCL-SF), from the MoBa cohort study (Version 8 of the quality assured data files; Magnus et al., 2016). Due to missing data on the SCL-SF (N = 7), the final sample of the supplementary analyses including SCL totaled 85 participants. The average level of prior symptoms was low (*M* = .68, *SD* = .68), with the majority of women (*N* = 76, 89.4%) reporting symptoms below, and approximately 10.6% (*N* = 9) reporting symptoms above the cut-off at 1.75.

To assess the impact of prior maternal symptoms of anxiety and depression on later child behaviors, we first ran a set of bivariate correlations between maternal prior and concurrent symptoms, child inhibitory control, and child internalizing and externalizing behaviors (for details, see Table 1). Next, we ran a two sets of hierarchical regression analysis—one for each outcome measure. In the first set, we examine whether children’s scores of inhibitory control moderates the observed association between prior maternal symptoms of anxiety and depression and child internalizing and externalizing behaviors. In the second set, we assess the moderating role of child inhibitory control on the association between concurrent maternal depressive symptoms and child internalizing behaviors, now controlling for prior maternal symptoms of anxiety and depression.

To explore the impact of other demographic variables such as gender, maternal education, and relationship status, we ran a set of bivariate correlations between these three variables and the initially included variables (maternal prior and concurrent symptoms, child inhibitory control, and child internalizing and externalizing behaviors) (see Table 1). Next, we ran a two sets of hierarchical regression analysis for each of the new predictor variables—one for each outcome measure (see Tables 6-11).

***Preliminary analysis***

As expected, prior maternal symptoms of anxiety and depression were significantly correlated with child internalizing and externalizing behaviors, but not with child inhibitory control. However, based on theoretical assumptions, child inhibitory control may still have a moderating effect on the association between prior maternal symptoms and child internalizing or externalizing behaviors, despite having no direct association with the outcomes. Prior and concurrent maternal symptoms were also significantly correlated, and maternal education was significantly positively correlated with prior maternal symptoms, as well as with child internalizing an externalizing behavior. Child gender was not correlated with either of the other variables, and relationship status was only negatively correlated with maternal education level—indicating that the women who were in a relationship at time of assessment had a lower education level compared to women who were categorized as single or divorced (see Table 1 below).

**Table 1.** Correlations between maternal symptoms, child outcomes, gender, maternal education, and relationship status.

|  |  |  | **1** | **2** | **3** | **4** | **5** | **6** | **7** |
| --- | --- | --- | --- | --- | --- | --- | --- | --- | --- |
| **1** | **Prior maternal symptoms of depression and anxiety** | *r* | 1 |  |  |  |  |  |  |
|  |  | *p* | . |  |  |  |  |  |  |
|  |  | *CI* | . |  |  |  |  |  |  |
| **2** | **Concurrent maternal depressive symptoms** | *r* | .64 |  |  |  |  |  |  |
|  |  | *p* | <.001 |  |  |  |  |  |  |
|  |  | *CI* | [.50, .75] |  |  |  |  |  |  |
| **3** | **Child internalizing behavior** | *r* | .35 | .44 |  |  |  |  |  |
|  |  | *p* | <.001 | <.001 |  |  |  |  |  |
|  |  | *CI* | [.15, .52] | [.26, .59] |  |  |  |  |  |
| **4** | **Child externalizing behavior** | *r* | .47 | .37 | .72 |  |  |  |  |
|  |  | *p* | <.001 | <.001 | <.001 |  |  |  |  |
|  |  | *CI* | [.28, .62] | [.12, .54] | [.60, .81] |  |  |  |  |
| **5** | **Child inhibitory control** | *r* | .07 | -.05 | .09 | -.09 |  |  |  |
|  |  | *p* | .554 | .642 | .379 | .387 |  |  |  |
|  |  | *CI* | [-.15, .27] | [-.25, .16] | [-.11, .29] | [-.29, .12] |  |  |  |
| **6** | **Child gender** | *r* | -.08 | .01 | .15 | -.03 | .45 |  |  |
|  |  | *p* | .460 | .948 | .144 | .813 | .294 |  |  |
|  |  | *CI* | [-.29, .13] | [-.20, .21] | [-.05, .35] | [-.18, .23] | [-.13, .28] |  |  |
| **7** | **Maternal education** | *r* | -.32 | -.35 | -.35 | -.334 | .12 | .75 |  |
|  |  | *p* | .004 | <.001 | <.001 | .002 | .281 | .698 |  |
|  |  | *CI* | [-.50, -.10] | [-.52, - 15] | [-.52, -.15] | [-.51, -.13] | [-.10, .32] | [-.18, .25] |  |
| **8** | **Relationship status** | *r* | -.20 | -.17 | -.19 | -.07 | -.10 | -.25 | .17 |
|  |  | *p* | .070 | .113 | .071 | .517 | .350 | .018 | .117 |
|  |  | *CI* | [-.40, -.02] | [-.37, -.04] | [-.38, .02] | [-.28, .14] | [-.30, .11] | [-.44, -.04] | [-.04, -.38] |

**Note.** Correlations (r) reflect Pearson correlation coefficient, with significance (p) determined using 2-tailed tests, and 95% confidence intervals (CI [LL, UL]) based on Fisher’s r-to-z transformation.

***Prior maternal symptoms of anxiety and depression: Internalizing behaviors***

Assessing the moderating role of child inhibitory control on the association between prior maternal symptoms of anxiety and depression and child internalizing behaviors, we ran a hierarchical regression analysis with prior maternal depressive symptoms entered first as the main predictor of child outcomes. As expected based on the bivariate correlations, this revealed an overall significant model *F*(1,83) = 11.65, *p* < .001, with prior maternal symptoms of anxiety and depression explaining 12.3% of the variance in child internalizing behaviors. Increasing levels of prior maternal symptoms of anxiety and depression is associated with an increase in child internalizing behaviors (see Table 2, Model 1). As a second step, after controlling for prior maternal symptoms of anxiety and depression in step one, child inhibitory control was added to the model. While the model as a whole remained significant (Table 2, Model 2), child inhibitory control did not significantly increase the model’s explained variance, *F*_Δ_(1,82) = .37, *p* = .543, *R^2^*_Δ_ = .004. Finally, we added the interaction term (Prior maternal symptoms X Child inhibitory control) as a third step. This did not significantly increase the model’s explained variance, *F*_Δ_(1,81) = .43, *p* = .512,  *R^2^*_Δ_ = .005, although the overall model remained significant (Table 2, Model 3).

**Table 2.** Hierarchical regression models testing the moderating effect of child inhibitory control on the association between prior maternal symptoms of anxiety and depression and child internalizing behaviors.

|  |  | **Model 1**  **(*β* [SE], *p*)** |  | **Model 2**  **(*β* [SE], *p*)** |  | **Model 3**  **(*β* [SE], *p*)** |
| --- | --- | --- | --- | --- | --- | --- |
|  |  |  |  |  |  |  |
|  |  |  |  |  |  |  |
| Prior maternal symptoms of anxiety and depression |  | .35 [.10], <.001 |  | .35 [.10], <.001 |  | .36 [.10], <.001 |
|  |  |  |  |  |  |  |
| Child inhibitory control |  |  |  | .06 [.10], .543 |  | .11 [.12], .385 |
|  |  |  |  |  |  |  |
| Prior maternal symptoms X  Child inhibitory control |  |  |  |  |  | .09 [.12], .512 |
|  |  |  |  |  |  |  |
|  |  |  |  |  |  |  |
| *R^2^* |  | .123 |  | .127 |  | .132 |
| Model df |  | 83 |  | 82 |  | 81 |
| Model *F* |  | 11.65 |  | 5.96 |  | 4.09 |
| *p* |  | <.001 |  | .004 |  | .009 |
|  |  |  |  |  |  |  |
|  |  |  |  |  |  |  |

***Prior maternal symptoms of anxiety and depression: Externalizing behaviors***

Assessing the moderating role of child inhibitory control on the association between prior maternal symptoms of anxiety and depression and child externalizing behaviors, we ran a hierarchical regression analysis with prior maternal symptoms of anxiety and depression entered first as the main predictor of child outcomes. As expected, this revealed an overall significant model *F*(1,83) = 23.35, *p* < .001, with prior maternal symptoms of anxiety and depression explaining 22% of the variance in child internalizing behaviors. Increasing levels of prior maternal symptoms of anxiety and depression is associated with an increase in child internalizing behaviors (see Table 3, Model 1). As a second step, after controlling for prior maternal symptoms of anxiety and depression in step one, child inhibitory control was added to the model. While the model as a whole remained significant (Table 3, Model 2), child inhibitory control did not significantly increase the model’s explained variance, *F*_Δ_(1,82) = 2.23, *p* = .139, *R^2^*_Δ_ = .021. Finally, we added the interaction term (Prior maternal symptoms X Child inhibitory control) as a third step. This did not significantly increase the model’s explained variance, *F*_Δ_(1,81) = .36, *p* = .549,  *R^2^*_Δ_ = .003, although the overall model remained significant (Table 3, Model 3).

**Table 3.** Hierarchical regression models testing the moderating effect of child inhibitory control on the association between prior maternal depressive symptom levels and child externalizing behaviors.

|  |  | **Model 1**  **(*β* [SE], *p*)** |  | **Model 2**  **(*β* [SE], *p*)** |  | **Model 3**  **(*β* [SE], *p*)** |
| --- | --- | --- | --- | --- | --- | --- |
|  |  |  |  |  |  |  |
|  |  |  |  |  |  |  |
| Prior maternal symptoms of anxiety and depression |  | .47 [.09], <.001 |  | .48 [.09], <.001 |  | .49 [.10], <.001 |
|  |  |  |  |  |  |  |
| Child inhibitory control |  |  |  | -.14 [.09], .139 |  | -.10 [.12], .377 |
|  |  |  |  |  |  |  |
| Prior maternal symptoms X  Child inhibitory control |  |  |  |  |  | .07 [.11], .549 |
|  |  |  |  |  |  |  |
|  |  |  |  |  |  |  |
| *R^2^* |  | .220 |  | .240 |  | .244 |
| Model df |  | 83 |  | 82 |  | 81 |
| Model *F* |  | 23.34 |  | 12.96 |  | 8.70 |
| *p* |  | <.001 |  | <.001 |  | <.001 |
|  |  |  |  |  |  |  |
|  |  |  |  |  |  |  |

***Concurrent maternal depressive symptoms, controlling for prior symptoms of anxiety and depression: Internalizing behaviors***

As presented in the main manuscript, child inhibitory control moderated the association between concurrent maternal depressive symptoms and child internalizing behaviors. To control for the potential impact of prior maternal symptoms of anxiety and depression on the observed interaction, we ran a hierarchical regression analysis with prior maternal symptoms of anxiety and depression entered as a first step to this model (Table 4, Model 1). As a second step, concurrent maternal depressive symptoms was added to the model, increasing the model’s explained variance, *F*_Δ_(1,82) = 9.37, *p* .003, *R^2^*_Δ_ = .090, and with the model as a whole remaining significant (Table 4, Model 2). As a third step, child inhibitory control was added to the model. While the model as a whole remained significant (Table 4, Model 3), child inhibitory control did not significantly increase the model’s explained variance, *F*_Δ_(1,81) = 1.02, *p* = .316, *R^2^*_Δ_ = .010. Finally, we added the interaction term (Concurrent maternal depressive symptoms X Child inhibitory control) as a fourth step. This significantly increased the model’s explained variance, *F*_Δ_(1,80) = 6.55, *p* = .012,  *R^2^*_Δ_ = .059, with the overall model remaining significant (Table 4, Model 4).

**Table 4.** Hierarchical regression models testing the moderating effect of child inhibitory control on the association between concurrent maternal depressive symptom levels and child internalizing behaviors, controlling for prior maternal symptoms of anxiety and depression.

|  |  | **Model 1**  **(*β* [SE], *p*)** |  | **Model 2**  **(*β* [SE], *p*)** |  | **Model 3**  **(*β* [SE], *p*)** |  | **Model 4**  **(*β* [SE], *p*)** |
| --- | --- | --- | --- | --- | --- | --- | --- | --- |
|  |  |  |  |  |  |  |  |  |
|  |  |  |  |  |  |  |  |  |
| Prior maternal symptoms of anxiety and depression |  | .35 [.10], <.001 |  | .10 [.12], .438 |  | .08 [.13], .520 |  | .10 [.12], .433 |
|  |  |  |  |  |  |  |  |  |
| Concurrent maternal depressive symptoms |  |  |  | .39 [.12], .003 |  | .41 [.12], .002 |  | .36 [.12], .006 |
|  |  |  |  |  |  |  |  |  |
| Child inhibitory control |  |  |  |  |  | .10 [.10], .316 |  | .25 [.11], .030 |
|  |  |  |  |  |  |  |  |  |
| Concurrent maternal depressive symptoms X  Child inhibitory control |  |  |  |  |  |  |  | -.29 [.08], .012 |
|  |  |  |  |  |  |  |  |  |
|  |  |  |  |  |  |  |  |  |
| *R^2^* |  | .123 |  | .213 |  | .223 |  | .282 |
| Model df |  | 83 |  | 82 |  | 81 |  | 80 |
| Model *F* |  | 11.65 |  | 11.09 |  | 7.74 |  | 7.84 |
| *p* |  | <.001 |  | <.001 |  | <.001 |  | <.001 |
|  |  |  |  |  |  |  |  |  |
|  |  |  |  |  |  |  |  |  |

***Concurrent maternal depressive symptoms, controlling for prior symptoms of anxiety and depression: Externalizing behaviors***

As presented in the main manuscript, child inhibitory control moderated the association between concurrent maternal depressive symptoms and child externalizing behaviors. To control for the potential impact of prior maternal symptoms of anxiety and depression on the observed interaction, we ran a hierarchical regression analysis with prior maternal symptoms of anxiety and depression entered as a first step to this model (Table 5, Model 1). As a second step, concurrent maternal depressive symptoms was added to the model. While the model as a whole remained significant (Table 5, Model 2), concurrent maternal depressive symptoms did not significantly increase the model’s explained variance, *F*_Δ_(1,82) = 1.57, *p* = .215, *R^2^*_Δ_ = .015, and with the model as a whole remaining significant (Table 5, Model 2). As a third step, child inhibitory control was added to the model. While the model as a whole still remained significant (Table 5, Model 3), child inhibitory control did not significantly increase the model’s explained variance, *F*_Δ_(1,81) = 1.84, *p* = .178, *R^2^*_Δ_ = .017. Finally, we added the interaction term (Concurrent maternal depressive symptoms X Child inhibitory control) as a fourth step. This significantly increased the model’s explained variance, *F*_Δ_(1,80) = 8.86, *p* = .004,  *R^2^*_Δ_ = .075, with the overall model also remaining significant (Table 5, Model 4).

**Table 5.** Hierarchical regression models testing the moderating effect of child inhibitory control on the association between concurrent maternal depressive symptom levels and child externalizing behaviors, controlling for prior maternal symptoms of anxiety and depression.

|  |  | **Model 1**  **(*β* [SE], *p*)** |  | **Model 2**  **(*β* [SE], *p*)** |  | **Model 3**  **(*β* [SE], *p*)** |  | **Model 4**  **(*β* [SE], *p*)** |
| --- | --- | --- | --- | --- | --- | --- | --- | --- |
|  |  |  |  |  |  |  |  |  |
|  |  |  |  |  |  |  |  |  |
| Prior maternal symptoms of anxiety and depression |  | .47 [.09], <.001 |  | .37 [.12], .005 |  | .39 [.12], .003 |  | .41 [.12], .001 |
|  |  |  |  |  |  |  |  |  |
| Concurrent maternal depressive symptoms |  |  |  | .16 [.12], .215 |  | .14 [.12], .279 |  | .08 [.12], .497 |
|  |  |  |  |  |  |  |  |  |
| Child inhibitory control |  |  |  |  |  | -.13 [.10], .178 |  | .03 [.11], .759 |
|  |  |  |  |  |  |  |  |  |
| Concurrent maternal depressive symptoms X  Child inhibitory control |  |  |  |  |  |  |  | -.32 [.08], .004 |
|  |  |  |  |  |  |  |  |  |
|  |  |  |  |  |  |  |  |  |
| *R^2^* |  | .220 |  | .234 |  | .251 |  | .326 |
| Model df |  | 83 |  | 82 |  | 81 |  | 80 |
| Model *F* |  | 23.35^***^ |  | 12.54^**^ |  | 9.06^***^ |  | 9.67^***^ |
| *p* |  | <.001 |  | <.001 |  | <.001 |  | <.001 |
|  |  |  |  |  |  |  |  |  |
|  |  |  |  |  |  |  |  |  |

***Concurrent maternal depressive symptoms, controlling for child gender: Internalizing behaviors***

To control for the potential impact of child gender on the moderating effect of child inhibitory control on the relationship between concurrent maternal depressive symptoms and child internalizing behaviors, we ran a hierarchical regression analysis with child gender entered as a first step to the original model, with gender coded as male (0) and female (1) (Table 6, Model 1). As expected, based on the lack of a direct correlation, this analysis did not reveal an overall significant model *F*(1,83) = 1.25, *p* = .267. As a second step, concurrent maternal depressive symptoms was added to the model, increasing the model’s explained variance, *F*_Δ_(1,82) = 21.29, *p* < .001, *R^2^*_Δ_ = .203, and with the model as a whole remaining significant (Table 6, Model 2). As a third step, child inhibitory control was added to the model. While the model as a whole remained significant (Table 6, Model 3), child inhibitory control did not significantly increase the model’s explained variance, *F*_Δ_(1,81) = 1.03, *p* = .313, *R^2^*_Δ_ = .010. Finally, we added the interaction term (Concurrent maternal depressive symptoms X Child inhibitory control) as a fourth step. This significantly increased the model’s explained variance, *F*_Δ_(1,80) = 7.84, *p* = .009,  *R^2^*_Δ_ = .063, with the overall model remaining significant (Table 6, Model 4).

**Table 6.** Hierarchical regression models testing the moderating effect of child inhibitory control on the association between concurrent maternal depressive symptom levels and child internalizing behaviors, controlling for child gender.

|  |  | **Model 1**  **(*β* [SE], *p*)** |  | **Model 2**  **(*β* [SE], *p*)** |  | **Model 3**  **(*β* [SE], *p*)** |  | **Model 4**  **(*β* [SE], *p*)** |
| --- | --- | --- | --- | --- | --- | --- | --- | --- |
|  |  |  |  |  |  |  |  |  |
|  |  |  |  |  |  |  |  |  |
| Child gender |  | .12 [.11], .267 |  | .10 [.10], .290 |  | .10 [.10], .333 |  | .12 [.09], .202 |
|  |  |  |  |  |  |  |  |  |
| Concurrent maternal depressive symptoms |  |  |  | .45 [.09], <.001 |  | .46 [.09], <.001 |  | .42 [.09], <.001 |
|  |  |  |  |  |  |  |  |  |
| Child inhibitory control |  |  |  |  |  | .10 [.10], .313 |  | .25 [.09], .026 |
|  |  |  |  |  |  |  |  |  |
| Concurrent maternal depressive symptoms X  Child inhibitory control |  |  |  |  |  |  |  | -.30 [.08], .009 |
|  |  |  |  |  |  |  |  |  |
|  |  |  |  |  |  |  |  |  |
| *R^2^* |  | .015 |  | .218 |  | .228 |  | .291 |
| Model df |  | 83 |  | 82 |  | 81 |  | 80 |
| Model *F* |  | 1.25 |  | 11.43 |  | 7.96 |  | 8.19 |
| *p* |  | .267 |  | <.001 |  | <.001 |  | <.001 |
|  |  |  |  |  |  |  |  |  |
|  |  |  |  |  |  |  |  |  |

***Concurrent maternal depressive symptoms, controlling for child gender: Externalizing behaviors***

Controlling for the potential impact of child gender on the moderating effect of child inhibitory control on the relationship between concurrent maternal depressive symptoms and child externalizing behaviors, we ran a hierarchical regression analysis with child gender entered as a first step to the original mode (Table 7, Model 1). As expected, based on the lack of a direct correlation, this analysis did not reveal an overall significant model *F*(1,83) = .09, *p* = .765. As a second step, concurrent maternal depressive symptoms was added to the model, increasing the model’s explained variance, *F*_Δ_(1,82) = 15.19, *p* < .001, *R^2^*_Δ_ = .156, and with the model as a whole remaining significant (Table 7, Model 2). As a third step, child inhibitory control was added to the model. While the model as a whole remained significant (Table 7, Model 3), child inhibitory control did not significantly increase the model’s explained variance, *F*_Δ_(1,81) = .79, *p* = .376, *R^2^*_Δ_ = .008. Finally, we added the interaction term (Concurrent maternal depressive symptoms X Child inhibitory control) as a fourth step. This significantly increased the model’s explained variance, *F*_Δ_(1,80) = 6.83, *p* = .011,  *R^2^*_Δ_ = .066, with the overall model remaining significant (Table 7, Model 4).

**Table 7.** Hierarchical regression models testing the moderating effect of child inhibitory control on the association between concurrent maternal depressive symptom levels and child externalizing behaviors, controlling for child gender.

|  |  | **Model 1**  **(*β* [SE], *p*)** |  | **Model 2**  **(*β* [SE], *p*)** |  | **Model 3**  **(*β* [SE], *p*)** |  | **Model 4**  **(*β* [SE], *p*)** |
| --- | --- | --- | --- | --- | --- | --- | --- | --- |
|  |  |  |  |  |  |  |  |  |
|  |  |  |  |  |  |  |  |  |
| Child gender |  | -.03 [.11], .765 |  | -.05 [.10], .634 |  | -.04 [.10], .690 |  | -.01 [.10], .891 |
|  |  |  |  |  |  |  |  |  |
| Concurrent maternal depressive symptoms |  |  |  | .40 [.09], <.001 |  | .39 [.10], <.001 |  | .35 [.09], <.001 |
|  |  |  |  |  |  |  |  |  |
| Child inhibitory control |  |  |  |  |  | -.09 [.10], .376 |  | .06 [.11], .581 |
|  |  |  |  |  |  |  |  |  |
| Concurrent maternal depressive symptoms X  Child inhibitory control |  |  |  |  |  |  |  | -.31 [.09], .011 |
|  |  |  |  |  |  |  |  |  |
|  |  |  |  |  |  |  |  |  |
| *R^2^* |  | .001 |  | .157 |  | .165 |  | .231 |
| Model df |  | 83 |  | 82 |  | 81 |  | 80 |
| Model *F* |  | .09 |  | 7.65 |  | 5.35 |  | 6.01 |
| *p* |  | .765 |  | <.001 |  | .002 |  | <.001 |
|  |  |  |  |  |  |  |  |  |
|  |  |  |  |  |  |  |  |  |

***Concurrent maternal depressive symptoms, controlling for maternal education: Internalizing behaviors***

Assessing the impact of maternal education level on the moderating effect of child inhibitory control on the relationship between concurrent maternal depressive symptoms and child internalizing behaviors, we ran a hierarchical regression analysis with maternal education entered as a first step to the original model, with maternal education coded as either basic schooling (0), or higher education (1) (Table 8, Model 1). Due to missing data on this variable, the total sample for these analyses is reduced to N = 80. As expected, based on the positive correlation, this analysis revealed an overall significant model *F*(1,78) = 5.86, *p* = .018. As a second step, concurrent maternal depressive symptoms was added to the model, increasing the model’s explained variance, *F*_Δ_(1,77) = 16.90, *p* < .001, *R^2^*_Δ_ = .167, and with the model as a whole remaining significant (Table 8, Model 2). As a third step, child inhibitory control was added to the model. While the model as a whole remained significant (Table 8, Model 3), child inhibitory control did not significantly increase the model’s explained variance, *F*_Δ_(1,76) = 1.64, *p* = .204, *R^2^*_Δ_ = .016. Finally, we added the interaction term (Concurrent maternal depressive symptoms X Child inhibitory control) as a fourth step. This significantly increased the model’s explained variance, *F*_Δ_(1,75) = 4.94, *p* = .029,  *R^2^*_Δ_ = .046, with the overall model remaining significant (Table 8, Model 4).

**Table 8.** Hierarchical regression models testing the moderating effect of child inhibitory control on the association between concurrent maternal depressive symptom levels and child internalizing behaviors, controlling for maternal education.

|  |  | **Model 1**  **(*β* [SE], *p*)** |  | **Model 2**  **(*β* [SE], *p*)** |  | **Model 3**  **(*β* [SE], *p*)** |  | **Model 4**  **(*β* [SE], *p*)** |
| --- | --- | --- | --- | --- | --- | --- | --- | --- |
|  |  |  |  |  |  |  |  |  |
|  |  |  |  |  |  |  |  |  |
| Maternal education |  | .26 [.11], .018 |  | .18 [.10], .085 |  | .19 [.10], .064 |  | .16 [.10], .116 |
|  |  |  |  |  |  |  |  |  |
| Concurrent maternal depressive symptoms |  |  |  | .42 [.10], <.001 |  | .42 [.10], <.001 |  | .39 [.09], <.001 |
|  |  |  |  |  |  |  |  |  |
| Child inhibitory control |  |  |  |  |  | .13 [.10], .204 |  | .26 [.11], .026 |
|  |  |  |  |  |  |  |  |  |
| Concurrent maternal depressive symptoms X  Child inhibitory control |  |  |  |  |  |  |  | -.26 [.08], .029 |
|  |  |  |  |  |  |  |  |  |
|  |  |  |  |  |  |  |  |  |
| *R^2^* |  | .070 |  | .237 |  | .253 |  | .300 |
| Model df |  | 78 |  | 77 |  | 76 |  | 75 |
| Model *F* |  | 5.86 |  | 11.97 |  | 8.60 |  | 8.02 |
| *p* |  | .018 |  | <.001 |  | <.001 |  | <.001 |
|  |  |  |  |  |  |  |  |  |
|  |  |  |  |  |  |  |  |  |

***Concurrent maternal depressive symptoms, controlling for maternal education: Externalizing behaviors***

To test the potential impact of maternal education level on the moderating effect of child inhibitory control on the relationship between concurrent maternal depressive symptoms and child externalizing behaviors, we ran a hierarchical regression analysis with maternal education entered as a first step to the original model, with maternal education coded as either basic schooling (0), or higher education (1) (Table 9, Model 1). As expected, based on the positive correlation, this analysis revealed an overall significant model *F*(1,78) = 5.27, *p* = .024. As a second step, concurrent maternal depressive symptoms was added to the model, increasing the model’s explained variance, *F*_Δ_(1,77) = 11.50, *p* = .001, *R^2^*_Δ_ = .122, and with the model as a whole remaining significant (Table 9, Model 2). As a third step, child inhibitory control was added to the model. While the model as a whole remained significant (Table 9, Model 3), child inhibitory control did not significantly increase the model’s explained variance, *F*_Δ_(1,76) = .54, *p* = .463, *R^2^*_Δ_ = .006. Finally, we added the interaction term (Concurrent maternal depressive symptoms X Child inhibitory control) as a fourth step. This significantly increased the model’s explained variance, *F*_Δ_(1,75) = 5.65, *p* = .020,  *R^2^*_Δ_ = .057, with the overall model remaining significant (Table 9, Model 4).

**Table 9.** Hierarchical regression models testing the moderating effect of child inhibitory control on the association between concurrent maternal depressive symptom levels and child externalizing behaviors, controlling for maternal education.

|  |  | **Model 1**  **(*β* [SE], *p*)** |  | **Model 2**  **(*β* [SE], *p*)** |  | **Model 3**  **(*β* [SE], *p*)** |  | **Model 4**  **(*β* [SE], *p*)** |
| --- | --- | --- | --- | --- | --- | --- | --- | --- |
|  |  |  |  |  |  |  |  |  |
|  |  |  |  |  |  |  |  |  |
| Maternal education |  | .25 [.11], .024 |  | .17 [.10], .096 |  | .17 [.10], .115 |  | .13 [.10], .202 |
|  |  |  |  |  |  |  |  |  |
| Concurrent maternal depressive symptoms |  |  |  | .36 [.10], .001 |  | .36 [.10], .001 |  | .32 [.10], .002 |
|  |  |  |  |  |  |  |  |  |
| Child inhibitory control |  |  |  |  |  | -.08 [.10], .463 |  | .07 [.12], .575 |
|  |  |  |  |  |  |  |  |  |
| Concurrent maternal depressive symptoms X  Child inhibitory control |  |  |  |  |  |  |  | -.29 [.09], .020 |
|  |  |  |  |  |  |  |  |  |
|  |  |  |  |  |  |  |  |  |
| *R^2^* |  | .063 |  | .185 |  | .191 |  | .247 |
| Model df |  | 78 |  | 77 |  | 76 |  | 75 |
| Model *F* |  | 5.27 |  | 8.74 |  | 5.97 |  | 6.16 |
| *p* |  | .024 |  | <.001 |  | .001 |  | <.001 |
|  |  |  |  |  |  |  |  |  |
|  |  |  |  |  |  |  |  |  |

***Concurrent maternal depressive symptoms, controlling for relationship status: Internalizing behaviors***

Exploring the impact of relationship status on the moderating effect of child inhibitory control on the relationship between concurrent maternal depressive symptoms and child internalizing behaviors, we ran a hierarchical regression analysis with relationship status entered as a first step to the original model, coded as either single/divorced (0), or married/cohabitant (1) (Table 10, Model 1). Due to missing data on this variable, the total sample for these analyses is reduced to N = 81. As expected, based on the lack of correlation with the dependent variable, this analysis did not reveal an overall significant model at the first step *F*(1,79) = .34, *p* = .562. As a second step, concurrent maternal depressive symptoms was added to the model, significantly increasing the model’s explained variance, *F*_Δ_(1,78) = 20.27, *p* < .001, *R^2^*_Δ_ = .205, and with the model as a whole now significant (Table 10, Model 2). As a third step, child inhibitory control was added to the model. While the model as a whole remained significant (Table 10, Model 3), child inhibitory control did not significantly increase the model’s explained variance, *F*_Δ_(1,77) = 1.21, *p* = .275, *R^2^*_Δ_ = .012. Finally, we added the interaction term (Concurrent maternal depressive symptoms X Child inhibitory control) as a fourth step. This significantly increased the model’s explained variance, *F*_Δ_(1,76) = 6.01, *p* = .017,  *R^2^*_Δ_ = .057, with the overall model remaining significant (Table 10, Model 4).

**Table 10.** Hierarchical regression models testing the moderating effect of child inhibitory control on the association between concurrent maternal depressive symptom levels and child internalizing behaviors, controlling for relationship status.

|  |  | **Model 1**  **(*β* [SE], *p*)** |  | **Model 2**  **(*β* [SE], *p*)** |  | **Model 3**  **(*β* [SE], *p*)** |  | **Model 4**  **(*β* [SE], *p*)** |
| --- | --- | --- | --- | --- | --- | --- | --- | --- |
|  |  |  |  |  |  |  |  |  |
|  |  |  |  |  |  |  |  |  |
| Relationship status |  | -.06 [.13], .562 |  | .05 [.12], .499 |  | .06 [.12], .578 |  | .06 [.12], .577 |
|  |  |  |  |  |  |  |  |  |
| Concurrent maternal depressive symptoms |  |  |  | .47 [.10], <.001 |  | .48 [.10], <.001 |  | .44 [.10], <.001 |
|  |  |  |  |  |  |  |  |  |
| Child inhibitory control |  |  |  |  |  | .11 [.10], .275 |  | .26 [.11], .028 |
|  |  |  |  |  |  |  |  |  |
| Concurrent maternal depressive symptoms X  Child inhibitory control |  |  |  |  |  |  |  | -.28 [.09], .017 |
|  |  |  |  |  |  |  |  |  |
|  |  |  |  |  |  |  |  |  |
| *R^2^* |  | .004 |  | .210 |  | .222 |  | .279 |
| Model df |  | 79 |  | 78 |  | 77 |  | 76 |
| Model *F* |  | .339 |  | 10.34 |  | 7.34 |  | 7.35 |
| *p* |  | .562 |  | <.001 |  | <.001 |  | <.001 |
|  |  |  |  |  |  |  |  |  |
|  |  |  |  |  |  |  |  |  |

***Concurrent maternal depressive symptoms, controlling for maternal education: Externalizing behaviors***

To test the potential impact of relationship status on the moderating effect of child inhibitory control on the relationship between concurrent maternal depressive symptoms and child externalizing behaviors, we ran a hierarchical regression analysis with relationship status entered as a first step to the original model (Table 11, Model 1). Due to missing data on this variable, the total sample for these analyses is reduced to N = 81. As expected, based on the lack of direct correlation, this analysis did not reveal an overall significant model *F*(1,79) = .13, *p* = .720. As a second step, concurrent maternal depressive symptoms was added to the model, increasing the model’s explained variance, *F*_Δ_(1,78) = 14.54, *p* < .001, *R^2^*_Δ_ = .157, and with the model as a whole now becoming significant (Table 11, Model 2). As a third step, child inhibitory control was added to the model. While the model as a whole remained significant (Table 11, Model 3), child inhibitory control did not significantly increase the model’s explained variance, *F*_Δ_(1,77) = .77, *p* = .384, *R^2^*_Δ_ = .008. Finally, we added the interaction term (Concurrent maternal depressive symptoms X Child inhibitory control) as a fourth step. This significantly increased the model’s explained variance, *F*_Δ_(1,76) = 6.36, *p* = .012,  *R^2^*_Δ_ = .067, with the overall model remaining significant (Table 11, Model 4).

**Table 11.** Hierarchical regression models testing the moderating effect of child inhibitory control on the association between concurrent maternal depressive symptom levels and child externalizing behaviors, controlling for relationship status.

|  |  | **Model 1**  **(*β* [SE], *p*)** |  | **Model 2**  **(*β* [SE], *p*)** |  | **Model 3**  **(*β* [SE], *p*)** |  | **Model 4**  **(*β* [SE], *p*)** |
| --- | --- | --- | --- | --- | --- | --- | --- | --- |
|  |  |  |  |  |  |  |  |  |
|  |  |  |  |  |  |  |  |  |
| Relationship status |  | -.04 [.13], .720 |  | .06 [.13], .565 |  | .06 [.13], .599 |  | .06 [.12], .598 |
|  |  |  |  |  |  |  |  |  |
| Concurrent maternal depressive symptoms |  |  |  | .41 [.10], <.001 |  | .40 [.10], <.001 |  | .36 [.10], <.001 |
|  |  |  |  |  |  |  |  |  |
| Child inhibitory control |  |  |  |  |  | -.09 [.10], .384 |  | .07 [.12], .576 |
|  |  |  |  |  |  |  |  |  |
| Concurrent maternal depressive symptoms X  Child inhibitory control |  |  |  |  |  |  |  | -.31 [.09], .012 |
|  |  |  |  |  |  |  |  |  |
|  |  |  |  |  |  |  |  |  |
| *R^2^* |  | .002 |  | .158 |  | .167 |  | .234 |
| Model df |  | 79 |  | 78 |  | 77 |  | 76 |
| Model *F* |  | .13 |  | 7.35 |  | 5.14 |  | 5.79 |
| *p* |  | .720 |  | .001 |  | .003 |  | <.001 |
|  |  |  |  |  |  |  |  |  |
